# In-silico computational approaches to study microbiota impacts on diseases and pharmacotherapy

**DOI:** 10.1186/s13099-023-00535-2

**Published:** 2023-03-07

**Authors:** Hassan Shokri Garjan, Yadollah Omidi, Mehdi Poursheikhali Asghari, Reza Ferdousi

**Affiliations:** 1 Department of Health Information Technology, School of Management and Medical Informatics, Tabriz University of Medical Sciences, Tabriz, Iran; 2 Department of Pharmaceutical Sciences, Nova Southeastern University, College of Pharmacy, Fort Lauderdale, FL USA; 3 Biotechnology Research Center, Tabriz University of Medical Sciences, Tabriz, Iran

**Keywords:** Microbiota, Microbiome, Microbe–disease prediction, Microbe–disease similarity, Similarity calculation method, Microbe–disease associations

## Abstract

Microorganisms have been linked to a variety of critical human disease, thanks to advances in sequencing technology and microbiology. The growing recognition of human microbe–disease relationships provides crucial insights into the underlying disease process from the perspective of pathogens, which is extremely useful for pathogenesis research, early diagnosis, and precision medicine and therapy. Microbe-based analysis in terms of diseases and related drug discovery can predict new connections/mechanisms and provide new concepts. These phenomena have been studied via various in-silico computational approaches. This review aims to elaborate on the computational works conducted on the microbe–disease and microbe–drug topics, discuss the computational model approaches used for predicting associations and provide comprehensive information on the related databases. Finally, we discussed potential prospects and obstacles in this field of study, while also outlining some recommendations for further enhancing predictive capabilities.

## Introduction

The gut microbiota is a collection of microorganisms that live in the mammalian gastrointestinal tract (GIT). This microbial population has a host-specific composition that changes with time and is susceptible to both exogenous and endogenous alterations [1]. Unlike the host genomic profile, the gut microbiome is a changeable environment that can be achieved with probiotics, prebiotics, nutrition, and community replacement techniques like fecal microbiota transplant [2]. The majority of these microbes live in the gastrointestinal tract, most abundant in the distal portion of the intestine. They synthesize essential amino acids, vitamins, and non-digestible components to aid in nutritional processes. Combined with host genetics, metabolic phenotypes can have a profound impact [3–7].

Environmentally, geography, diet, aging, the use of drugs and antibiotics, stress, and diseases can affect the gut microbiota [7–11]. A balance of microbiota is believed to protect the host body from physiological disorders. Based on a plethora of compelling evidence, there might be a correlation between the emergence of diseases and the changes in the composition and amount of microbiome in the body [12, 13]. Evidence suggests that changes in gut microbiota are linked to a variety of diseases and immune and metabolic dysfunctions, including hypertension, heart attack, myocardial infarction, stroke, coronary artery disease, diabetes, and chronic kidney disease [9, 14, 15]. Additionally, the human GIT microbiota, as a predictor of human health and the therapeutic response, can influence the reaction(s) of the body to a variety of treatments, from dietary and lifestyle modifications to drugs and surgical procedures [2]. It is deemed that the intestinal microbiota interacts with almost all human cells and is considered a major factor in host metabolism and also a new source of therapy [16].

A beneficial commensal or symbiotic relationship between the human and the microbiota of the body is proved by the advances in sequencing technology and recent bioinformatics discoveries [17, 18]. Meanwhile, some researchers develop and apply computer techniques to identify the effects of microbes on human disease. For instance, Coelho et al. have suggested a computational technique that takes into account the interaction between microbial and human proteins to anticipate the effect of microbial proteins on human biological processes [19]. The Human Microbiome Project started in 2007, is another well-known instance of a microbe project [20]. Finding Microbe–Disease Associations (MDA) might be extremely beneficial in areas that deal with diseases, such as medications and pathogenic genes [21]. The gut microbiota is now recognized as being responsible for adjusting many physiological functions of the host [25, 26]. Additionally, the identification of microbe–disease relationships offers several insights into the pathophysiology of disease. Notable computational techniques have recently been developed to investigate the influence of microbiota on human disease, and medications [30–35]. In this article, we strived to fully inspect the computational methods for predicting microbial disease associations, which can be divided into six categories:

I. Path-based methods: Path-based methods allow predictions in heterogeneous networks by calculating path-based scores between microbe nodes and disease nodes.
II. Methods based on Random Walks: A walker walks in a transfer likelihood network made up of microbe and disease nodes at random. These strategies look for a probable association by calculating the likelihood of a random walker completing a path that starts with a node on one side of the association and ends with a node on the other.
III. Bipartite Local Models (BLMs): Such methods compute Microbe–disease association (MDA) forecast scores from two viewpoints of diseases and microbes. The collective prediction scores on both sides are used to determine the final prediction ranking.
IV. Matrix factorization approaches: an interaction matrix is factorized into two low-dimensional matrices, one representing disease features and the other representing microbe ones. The final projected matrix is the sum of two low-dimensional matrices.
V. Machine learning-based: The machine learning-based method uses fewer parameters that can save time and achieve strong performance.
VI. Network-based methods: Network-based methods have used Graph Attention Network (GAT), MLP layers to predict new connections, automatic learning of a nonlinear function, and so on to predict new connections.
VII. Other methods: Certain methods may not be sorted into the groups above, but they are grouped as "other methods."

Additionally, various drugs can alter the structure and composition of the gut microbiome and thus change its biological function, such as the ability to metabolize. On the other hand, metabolism and drug outcomes may be influenced by microbial metabolic processes and their metabolites. Understanding the mutual relationship between drugs and microbiomes, as well as how it affects drug clinical outcomes, paves the way for next-generation interventions to reduce disease complications [22]. Little is known about the impact of the microbial gene pool on medications prescribed in various areas of the human body, as well as the impact of microbiome modifications on drug destiny, behavior, toxicity, and therefore a human reaction to care [23]. Remarkably, recent research has shown a solid connection between the microbiota and the pharmacological effects of chemotherapy [24] and immunotherapy [25, 26]. The microbial diversity in the body is intriguingly reduced, in large part due to the interaction of chemical drugs with the host immune system. As a result, the effect of such drug molecules might be decreased, and other consequences may occur too. The human microbiome, particularly the gut microbiome, improves the efficacy of chemo-drugs through digestion, enzyme degradation, ecological variations, and immunomodulatory. A recent study has taken advantage of the microbiome's role in shaping the effectiveness and toxicity of these chemotherapy agents [27]. The relationship between gut microbes and the currently used non-antibiotic drugs seems to be very complicated, in which drugs can affect the gut microbiome's makeup, and the gut microbiome can also enzymatically alter the drugs [28]. The individual's reaction to the medication may alter bioavailability, bioactivity, and/or toxicity, a phenomenon known as" pharmaco-microbiome" [29–31]. Variations in the human microbiome(i.e., the synthesis of human-associated microbial species and their genomes) might impact medication disposition, behavior, and toxicity, according to the concepts of pharmacy and toxico-microbiome [32].

The gut microbiota influences medication and cenobitic metabolism in both overt and indirect ways, which may affect effectiveness and toxicity [33]. Advances in gut microbiota modeling and research will expand our understanding of their function in health and disease, allowing for the customization of current and prospective medicinal and prophylactic modalities [34]. Moreover, various human infectious diseases are caused by an imbalance in microbial communities [20, 35]. GIT microbes also play an important role as a therapeutic target in precision medicine and modulation of drug activity or toxicity [36], while their diversity and function can be altered by drugs [37].

**Fig. 1:**
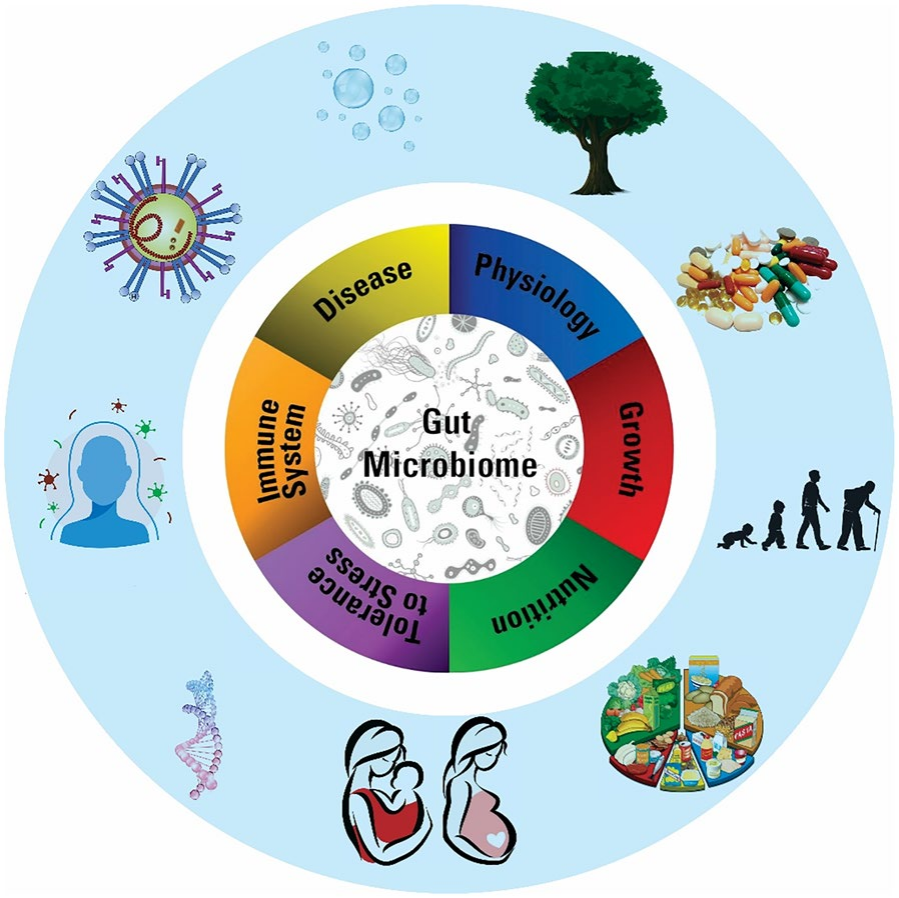
The human gut microbiota is a predictor of human health and therapeutic response. The gut microbiota influences a person's reaction to a variety of treatments, from dietary and lifestyle modifications to drugs and surgical procedures

Besides, with the increasing emergence of drug-resistant microbes, it is necessary to identify microbial-pharmacological associations in very large sizes [36]. For this purpose, several models have been proposed and designed to identify the association of medicinal microbes, including the Ensembling graph attention networks for predicting human microbe–drug association [38]. Based on the heterogeneous network embedding representation, the association mining method was used to detect microbe–drug interactions [39]. To compute potent associations between microbe and drug, Zou et al. developed a method based on the KATZ measure [40]. In another study, Long et al. proposed a computational Method based on a novel Graph Convolutional Network (GCN) framework for predicting before-mentioned associations [41]. These approaches can be summarized as follows:

- Neural Network (GCN): A neural network is a collection of algorithms that attempts to understand underlying associations in a set of input data using a procedure that mimics how the human brain works.
- Assembling a graph with attention function: In various graphs, each node (e.g., microbes, and drugs) can contain a variety of semantic knowledge. The attention function at the diagram level is used to efficiently collect node embeds from input diagrams, merge information, and remove noise from various diagrams.
- Heterogeneous network embedding representation: In this method, by combining Metapath2Vec with the recommendation of a two-part network, a heterogeneous embedded network demonstration framework is used to predict the association of microbes and drugs. To improve the prediction accuracy, the proposed bias bipartite network Embedding (BiNE) forecasting algorithm has been created and used.
- KATZ measurements: In this method, most of the heterogeneous network of medicinal microbes is created based on two similar networks and known connections of medicinal microbes. Based on these networks and KATZ measures, the process of predicting the potential relationships between drugs and microbes was performed. The human gut microbiota, as a predictor of human health and therapeutic response, is shown in Fig. 1.

## Prediction of microbiome association with drug and disease

In latest years, with the speedy development of strategies in bioinformatics and life science, a massive quantity of biomedical information has been amassed, based on which researchers have evolved numerous computational procedures to discover potential associations between human microbes, drugs and diseases. This article offers a thorough analysis of current developments in identifying possible relationships among microbes, drugs and diseases using biological data and computational models.

### Drug–microbe association

It should be noted that drugs can change the species diversity and function of microbial communities [36], and the number of drug-resistant bacteria is growing. In this line, microorganisms play a vital role in lowering the medications’ adverse reactions. Collectively, there is an urgent need to recognize the possible pharmaceutical-microbial associations [37]. In the rest of this section, the studies related to the prediction of microbe–drug relationships in the literature were reviewed.

#### Graph convolutional network (GCN)

Long, Y. et al. used various sources of biomedical information and created several networks (diagrams) for microbes and drugs. Then, they developed a novel ensemble framework of graph attention networks with a hierarchical attention mechanism for microbe–drug association prediction from the constructed multiple microbe–drug graphs, denoted as Ensembling graph attention networks for human microbe–drug association prediction (EGATMDA). Specifically, for each input graph, a graph convolution network is designed according to the node surface to learn to embed the nodes (e.g., microbes and drugs). To effectively integrate node embeds from multiple input diagrams, graph-level attention has been implemented to learn the importance of different input diagrams [38].

#### Graph attention networks

The proposed Graph Convolutional Network (GCN) based framework for predicting human Microbe–drug Associations (MDA), named GCNMDA is a convolutional neural network-based model for predicting drug-microbe interactions. Initially, a heterogeneous network is built to combine microbial gene information, drug chemical information, and microbe–drug interactions. Later, an RWR-based preprocessing mechanism is designed to extract effective properties. Finally, a CRF layer is generated in the GCN to enhance the learning of node representation for drugs and microbes so that similar nodes have similar representations. A layer of the CRF attention mechanism is designed to accurately collect representations from neighbors [42].

#### Heterogeneous network embedding representation

##### Adjacency matrix

In this approach, the information obtained from the confirmed experimental results related to human microbe–disease (microbe–drug) is extracted from the corresponding databases for microbe–disease (microbe–drug) associations. Then, an adjacency matrix A ∈ R^nd*nm^ is created (nd and nm show the number of diseases (drugs) and the number of microbes, respectively) as follows:

$$aij=\left\{\begin{array}{ll} 1 & if\,association\,between\,disease (drug)\,di\,and\,microbe\,mi\\ 0, & else\end{array}\right.$$

##### Similarity calculation and heterogeneous network

Various computational methods, that have been designed and proposed to predict microbe–disease (microbe–drug) data, are mentioned in the previous sections. The approaches can be classified into two groups: (i) those that use known disease-microbe relationships to calculate microbe–disease similarity, and (ii) those that use extra data.

In a method for determining similarity based on microbe–disease associations, the adjacent matrix A ∈ R^nd×nm^ is used as the input, and the similarity matrix between microbial S_m_ ∈ R^nm×nm^ and the similarity between S_d_ ∈ R^nd×nd^ disease is used as the output. The similarity calculation methods are the same for diseases-microbes (drugs) and the methods include Gaussian interaction profile kernel similarity [43]. The following approaches can be implemented:

*Cosine similarity:* In Euclidean space, the cosine similarity measures the cosine of the angle between two interaction profiles. Having capitalized on this approach, a few studies were able to obtain the microbe and disease similarity matrix [21, 44].

*Spearman correlation similarity*: Spearman correlation coefficients as similarity ratings are calculated using sequences of positions or time points of pairwise microbes [45].

In a recent study, Wang et al. advanced a gene-based disease association approach based on neighbor-dependent similarity estimation. In most studies, after creating similar networks for diseases and microbes, researchers have used known microbe–disease associations through databases to construct the proposed models [46].

Two researchers have proposed a biased two-way network algorithm to predict the most likely microbe–drug relationships and increase the accuracy of the proposed model. Heterogeneous Network Embedding Representation framework for Microbe Drug Association (HNERMDA) is based on the representation of an embedded heterogeneous network via metapath2vec and the recommendation of a two-part network. To build heterogeneous networks, they capitalized on interactions between microbes and drugs, such as drug-microbe interactions [39].

#### KATZ measurements

Using known drug-microbe associations, a microbe similarity network is constructed by calculating the GIP core similarity of microbes. Due to the two similar networks and similar connections of known medicinal microbes, a heterogeneous network of medicinal microbes is created. An HMDAKATZ model is designed to predict drug–microbe communication [40].

#### Multi-modal variational graph embedding

**Fig. 2:**
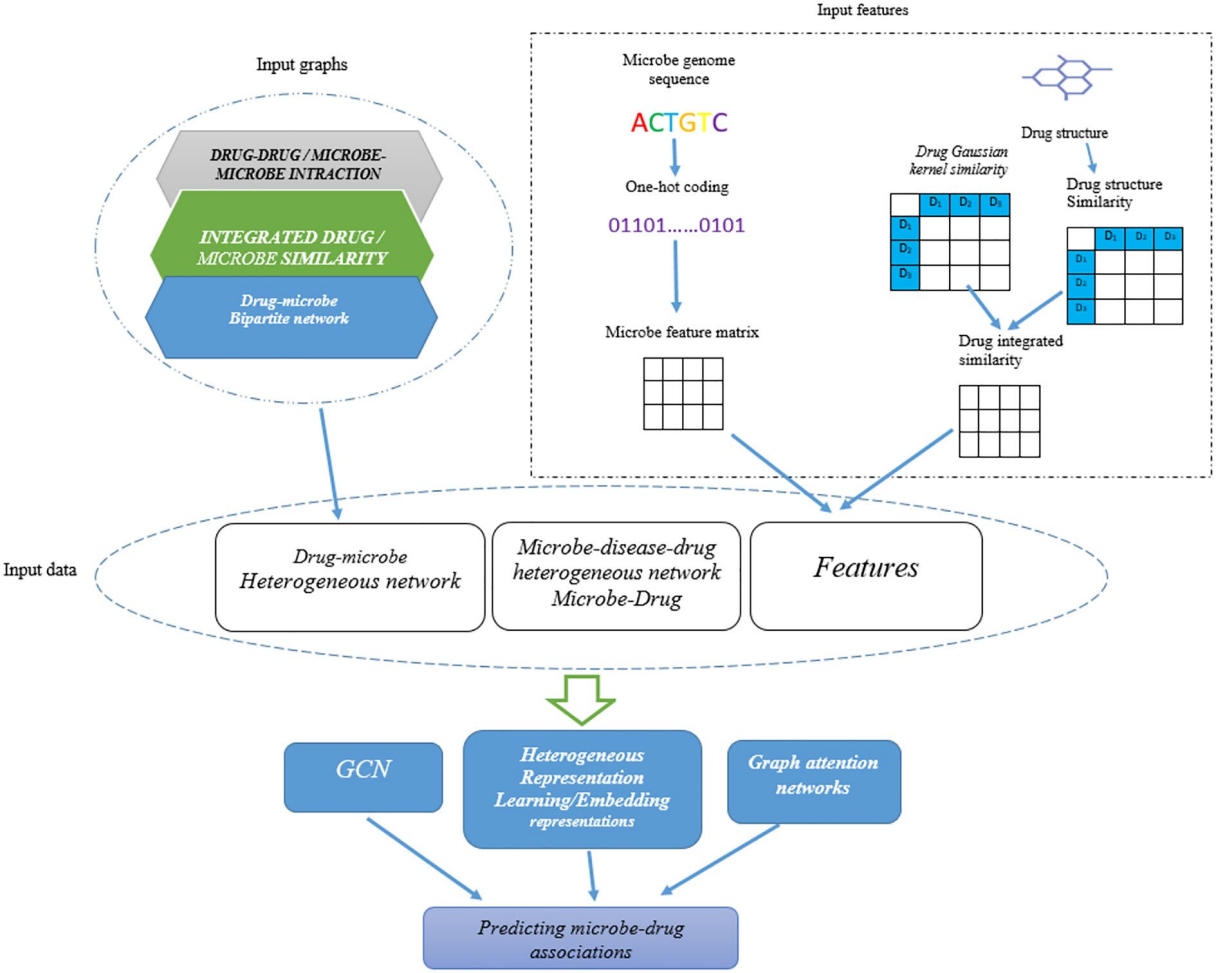
The architecture of predicting microbe–drug relationships using a convolutional neural network model

A multi-modal variational graph embedding model for predicting microbe–drug associations (Graph2MDA) is a new technology that uses a graph autoencoder to predict microbe–drug interactions variational graph auto encoder (VGAE). Created multi-modal attributed graphs based on molecular structures, microbe genetic sequences, and function annotations of bacteria and pharmaceuticals. A deep neural network classifier was used to predict microbe–drug relationships [47]. Figure 2 represents the architecture of predicting microbe–drug relationships using a convolutional neural network model.

#### Recruited datasets and approaches for prediction of microbe–drug associations

Previous studies on the microbe–drug relationship have used a variety of data.

Table 1 lists the data used to predict microbe–drug based on the information we reviewed.

**Table 1:** A list of all the data that fed into the microbe–drug association prediction

Data	Source	Original from	Size/coverage in MDAD	The process of similarity
MDAD	A database that gathers various types of data from different sources to discover the interaction between microbes and drugs and to promote the detection of antimicrobial drugs and the use of microbiomes in medicine (http://chengroup.cumt.edu.cn/MDAD)	Multiple prescription libraries and associated papers yielded 5,055 entries representing 1,388 medicines and 180 microbes	There are 5,055 entries in this collection of clinical or experimental support collaborations, including 180 microbes and 1,388 medications	After deleting duplicate information at the end of 2470, the relationship between 1373 drugs and 173 microbes has been used for the similarity process
Drug-Drug Interaction	DrugBank is one-of-a-kind bioinformatics and chemical informatics repository that integrates specific drug data with extensive drug target data. (https://www.drugbank.ca/releases/latest)	The latest version includes 14,522 drug imports, including 2,683 approved small-molecule drugs, 1,644 biology-approved drugs, 131 nutrients, and more than 6,654 experimental drugs	About 5249 extra protein sequences are associated with these drug inputs. Each input contains more than 200 pieces of data, half of which is dedicated to pharmaceuticals/Half of the data is related to chemical data, while the other half is related to target drug or protein data	Inputs such as drugs in MDAD Search have finally created a network of drug and drug interactions involving 5587 interactions with 181 drugs
Drug Structural Similarity Matrix a Biofilm Drug Virus	Chemical structure search servers are for network analysis and clarifying the relationship between the genomic and chemical importance of metabolic pathways: (http://www.genome.jp/tools/simcomp) The ‘a Biofilm’ (http://bioinfo.imtech.res.in/manojk/abiofilm/): There are three sub-categories in this category, including data visualization, a database, and a prediction module Lists the activities and stages of development of 118 drugs that target 83 human viruses (https://drugvirus.info/tech_doc/)	There are 5027 interactions between 1720 medications and 140 microorganisms in the database of anti-biofilm compounds, which includes gram-negative, gram-positive, and fungal microorganisms The database allows users to explore virus-BSAA (Broad-spectrum antiviral agents) interactions in real time. DrugVirus.info is a free tool that includes a feedback form on its website		SIMCOMP is a graph-based method for finding the maximum isomorphism of a common sub graph by finding the maximum number of clicks in the correlation diagram, and SUBCOMP is a broad method for solving the problem of sub graph isoforms
Human Drug Virus Database (HDVD)	• They devoted significant resources to collecting drug-virus interaction entries from the literature to create the Human Drug Virus Database (HDVD).SCPMF (similarity constrained probabilistic matrix factorization) is a novel technique for identifying new medication-virus interactions for therapeutic repurposing [68]			

In addition, different approaches for predicting the relationship between microbe–drug are summarized in Table 2.

**Table 2:** Different methods to predict microbe–drug association

Category	Method	Description	Refs.
Graph Convolutional Network (GCN)	GCNMDA	A conditional random context (CRF) and a CRF layer focus function in the hidden GCN layer are used to ensure that the same nodes have the same representations	[61]
Ensembling graph attention	EGATMDA	To learn embedded nodes for microbes and drugs, a convolutional graph network is built at the node level for each input diagram	[55]
Heterogeneous network embedding representation	HNERMDA	Metapath2vec has developed a heterogeneous network display learning approach for learning low-embedded microbe and drug displays in this context	[56]
Multi-modal variational graph embedding	Graph2MDA	A graph with variations A deep neural network classifier was used to predict microbe–drug relationships after an auto encoder was trained to learn the informative and interpretable latent representations of each node and the whole graph	[67]
Based on KATZ measurements	KATZ	By bringing the chemical structures together and the similarity of the nucleus, they have created the Gaussian interaction profile of the drug unification network	[57]

#### Comparison and application of models to each other

Since predicting the interactions between microbes and drugs is a new field of study, few computational approaches have been proposed for this critical task. The various approaches for link prediction problems in the field of bioinformatics and the existing techniques for microbe–drug interactions are compared [38, 48–50]. The Graph2MDA model had the highest AUC value, followed by LAGCN, while NTSHMDA had the lowest AUC value. Deep learning-based methods frequently outperform more traditional machine learning-based ones. The more effective method provides the following benefit over other models: Using multimodal feature graphs based on ontological information, multiple similarities between microbes and drugs, and their known relationships, methods may fully use many different sorts of features and links. Additionally, by incorporating the topological structure into multimodal feature networks, the impact of the cold start problem is lessened. Potentially mitigate the effect of similar noises [38].

### Microbe–disease association

According to some new research [51], microbes are being increasingly linked to human pathogens. Disease-related microbe research aims to understand disease processes and the creation of novel diagnostic and therapeutic methods. Many theoretical models for predicting disease-causing microbes have been suggested. In the continuation of this section, we review the studies related to the prediction of microbe disease relationships that exist in the literature.

#### Path-based methods

Weighted meta-graph-based model on heterogeneous information network (WMGHMDA) have been presented to predict the relationship between diseases and microbes. Path-based approaches examine indirect pathways across networks, which often evaluate the weight of a prospective route as the score of unknown relation. The Meta-Graph search algorithm is run on the heterogeneous network to count the meta-weight patterns of each disease-microbe pair. Summing the contribution values of the related weighted Meta graphs yields the likelihood score for each pair of disease-Microbe [52].

BWNMHMDA (Bidirectional Weighted Network model Human Microbe–disease Association Prediction) is a new method for predicting the microbe–disease association based on the Bidirectional Weight Network. The main idea of this model is to produce a bidirectional disease-microbe communication network that converts them into matrices to compute the probability of correlation. It can be achieved by assigning weight to nodes and edges in the integrated network using the similarity of the Gaussian interaction profile kernel [53].

The PBHMDA (Path-Based Human Microbe–disease Association Prediction) proposes a new path-based prediction model for inferring potential microbe–disease associations. It is based on the main similarity of Gaussian interaction profiles for diseases and Gaussian interactions between microbes. A special depth-first search algorithm was designed in the model to ensure no duplicate nodes were found [54].

The KATZ measurement model was proposed to predict the Human Microbe–disease Association (KATZHMDA) Researchers combined the number of walks and their distances as an appropriate measure index for measuring the possible interaction likelihood between microbes and diseases. It is based on the graph constructed by the established microbe–disease association network, microbe similarity network, and disease similarity network [48].

By integrating several data sources and path-based HeteSim scores, Fan et al. developed a new method for predicting disease-microbe Multiple Data sources and Path-based HeteSim scores for Human Microbe–disease Associations (MDPH_HMDA) communication. The similarity of microbes was calculated by combining microbial functional scores and Gaussian core profile similarity. The similarity of the disease pairs was calculated using the similarity scores based on the symptoms. The HeteSim method has been used to obtain the relevance score and normalized measurement from each disease-microbe pair [55].

#### Random walk methods

For iterative walking, random walk methods use a graph-based transfer likelihood matrix. Niu et al. made a higher-order hyper graph sample to accurately determine the intrinsic association between microbes and human diseases. They develop a model based on the random walk on hypergraph for microbe–disease association prediction (RWHMDA). They ranked all-volunteer microbes for every perused human disease. Hypergraphs can efficiently mitigate data loss occurring in the normal graph methodology. For the generated hypergraph, the core similarity of the Gaussian interaction profile, random walk, and integration of known microbe–disease associations from the HMDAD database was performed [56].

A heterogeneous network by combining the Gaussian interaction profile microbial similarity network and the Gaussian interaction profile disease similarity network has been produced by known networks of microbe–disease associations. Then, a novel way for predicting the future microbial and disease relationships based on extensive optimized random walking was announced by introducing network topological similarity (NTSHMDA) [49].

Zou et al. have combined the microbial similarity network and the disease similarity network to generate a heterogeneous network. A two-random walk algorithm was implemented on the network generated by the Gaussian interaction profile's similarity and logistic transformation. A novel computational model to predict potential microbe–disease associations by bi random walk on the heterogeneous network. Developed a new computational model for predicting potential human microbe–disease associations by bi random walk in heterogeneous network (BiRWHMDA) [57].

Zhang et al. proposed the bi-direction similarity integration label propagation (BDSILP) method for predicting microbe–disease associations. Using the Mesh, the semantic similarity of the disease and the functional similarity of the microbes were calculated. With the help of integrated disease similarity and integrated microbial similarity, they have produced two graphs. And BDSILP does the label propagation on the graphs to score the pairs of disease-microbe. BDSILP accepts the weighted mean of their scores as final predictions [58].

The symptom-based likeness is calculated by the concurrence of diseases and the term symptoms. After calculating the similarity of the core of the Gaussian interaction profile of microbes based on known microbial disease associations, the similarity with the logistic function was obtained. Using the Similarity Network Fusion (SNF) method with similarity based on symptoms and the similarity of the core, the Gaussian interaction profile was calculated according to the known microbe–disease associations of the disease network. The two networks created for microbes and disease have been combined by well-known microbe–disease associations and used by BRWMDA (Bi-random walk microbe–disease associations) to predict potential new microbe–disease relationships through random walking with different stages in microbial and disease networks [59].

After extracting information about the disease and germs, microbial networks were built using Spearman, and the disease network was generated based on the symptoms. Then, by combining the networks formed, a heterogeneous network of disease microbes is formed. Shen et al. developed the random walk with a restart algorithm for the heterogeneous network, using the goal disease and corresponding microbes as seed nodes. They employed this algorithm to reveal the latent relationship between diseases and microbes [60].

A team of researchers has proposed a new model of extended random walking with restart optimized by Particle Swarm Optimization (PRWHMDA) based on human microbe–disease associations. Wu et al. used cosine to calculate the similarity of diseases and microbes. Then, by combining networks, they formed a heterogeneous interconnected network. They introduced the RWR method to obtain strong communications [44].

Wang et al. have proposed a novel computational model based on the bidirectional label propagation to predict potential human microbe–disease associations (NBLPIHMDA). The Gaussian interaction profile kernel similarity was applied to measure the disease similarity matrix along with the microbe similarity matrix. The edge weights of nodes in these two networks were determined. Bidirectional mark dissemination was used to achieve the association score matrix between diseases and microbes [61].

Using known connections from microbial network databases, disease networks and microbe–disease networks were created. A heterogeneous network was constructed using known microbe–disease associations from the database, the microbial network, and the disease network. Wang et al. then predicted novel microbe–disease associations by a new method called the double ended restart random walk human microbe–disease association model (DRWHMDA) implemented on the interconnection network [62].

#### Bipartite local models

Fundamentally, the bipartite local models work independently on both sides of a microbe–disease pair and can be combined to provide a conclusive prediction outcome. These approaches are capable of making independent observations on both the microbe and the disease fronts. The final scoring matrix is based on the combination of the probability scores related to user-based and case-based collaborative filtering [63].

Zou et al. proposed a model using a combination of a neighborhood-based prediction model and a graph-based recommendation model for human microbe–disease association (called NGRHMDA). The graph-based prediction model presents a two-step diffusion approach on the microbe–disease bipartite graph. Two new integrated adjacent matrices have been developed based on the similarity of symptom-based diseases and on the similarity of Gaussian-based microbes to consider microbial and disease similarities [64].

The core similarity of the Gaussian interaction profile for germs and disease was extracted from the microbe–disease linkage network. Then, constructing and minimizing the cost function for optimal classifiers in microbe and disease space turned it into an integrated classification. A semi-supervised computational model_Laplacian Regularized Least Squares for Human Microbe–Disease Association (called LRLSHMDA) was proposed by Wang et al. to predict disease-microbe relationships [65]. Based on known microbe–disease communication networks, a heterogeneous network was created from the HMDAD database for the main similarity of disease Gaussian interaction profiles and microbe Gaussian interaction profiles. Then, Bao et al. planned the Network Consistency Projection for Human Microbe–disease Association prediction model (called NCPHMDA) to discover potential disease-microbe associations [66]. The KATZBNRA model, like the KATZHMDA, was designed by Li et al. using the KATZ criterion and the core similarity of the Gaussian interaction profile for diseases and microbes based on the known associations. In addition, they utilized a bipartite (two-way) Network Recommendation (BNR) algorithm to increase the prediction accuracy more than KATZHMDA [67].

#### Matrix factorization methods

The theory behind matrix factorization is that the input matrix decomposes into two low-dimensional matrices and the product of the two low-dimensional matrices approximates the input matrix [68, 69]. Wu et al. discovered disease characteristics by combining two similarities based on the Gaussian kernel and one based on symptoms. The microbial properties have also been calculated using the similarity of the Gaussian kernel. They presented a computational model using matrix completion to predict the association of the human microbe–disease profile (called MHMDA) [70]. Chen et al. introduced a method for predicting microbe–disease associations based on the Kernelized Bayesian Matrix Factorization (KBMF), which is dependent on the Gaussian interaction profile kernel similarity for microbes and diseases [71].To compute the microbial similarity and similarity of the disease, Liu et al. used the similarity of the core of the Gaussian interaction profile and applied logical functions to adjust the similarity of the disease. Based on the known microbe–disease associations, they suggested a model for predicting microbial disease associations using the regular non-negative matrix factorization chart (NMFMDA) [72].

By merging the known disease-microbe associations and the similarity of the core of the Gaussian interaction profile, Shen and his colleagues offered a Collaborative Matrix Factorization for Human Microbe–disease Association Prediction (CMFHMDA) model [73].

For the prediction of human microbe–disease associations, a novel predictive model of graph regularized non-negative matrix factorization (called GRNMFHMDA) was developed by He et al. Microbe and disease similarity were initially calculated using symptom-based disease similarity and Gaussian interaction profile kernel similarity for microbes and diseases, respectively. To prevent a negative effect on prediction results, a preprocessing phase was used in which unknown microbe–disease pairs were given associated probability scores. Finally, a graph-regularized non-negative matrix factorization method was employed to concurrently determine the possible correlations with all diseases [74]. Qu et al. introduced a statistical model of matrix decomposition and label propagation for the Human Microbe–disease Association prediction (so-called MDLPHMDA) by integrating proven microbe–disease associations obtained from the HMDAD database, disease symptom similarity, and Gaussian interaction profile kernel similarity for microbes and diseases. Using the spare learning method (SLM) on the original association details derived from HMDAD, a new adjacency matrix of microbe–disease associations was developed, and possible microbe–disease associations were further predicted using the label propagation algorithm (LPA) [75]. A Deep Matrix Factorization Prediction (DMFMDA) model has been proposed by Liu et al. to predict the associations between microbes and diseases that do not require microbial and disease-like networks and is based on deep neural networks, which combine the linear modeling advantages of matrix factorization with the non-linear modeling advantages of a multi-layer perceptron [76].

#### Network based methods

##### Graph attention networks

Long et al. present a new graph-attention network-based model for microbe–disease association prediction (called GATMDA) in a bipartite network, combining inductive matrix completion (IMC). Researchers used functional similarities of microbes, functional similarities of diseases, and Gaussian kernel similarities to obtain comprehensive specifications for microbes and diseases. Graphic Attention Networks (GAT) then introduced a GAT criterion for learning to display nodes using talking heads, which helps maintain a more informative display model [77].

Liu et al. proposed a multi-component Graph Attention Network based system to predict microbe -disease association (MGATMDA). By using a node-level attention mechanism, the decomposer first decomposes the edges in a bipartite network to discover the latent components. The combiner then automatically reassembles these hidden parts to provide a coherent embedding for component-level attention prediction. Finally, a fully linked network is employed to forecast known and unknown connections between bacteria and diseases [78].

##### Models based on neural networks

Using the similarity of microbial classification, the similarity of microbial interaction characteristics and disease interaction, semantic similarities and disease symptoms, and known disease and microbial associations, Ma et al. have developed a new method (NinimHMDA) based on neural integration of neighborhood information in a multiplex heterogeneous network (MHEN)for different types of human microbe–disease association prediction [79]. Li et al. proposed a new back-propagation neural network model to predict microbial-disease association (BPNNHMDA). The model input is a matrix of known microbe–disease associations, and its output is a matrix of potential microbe–disease association probabilities. An activation function is built based on the hyperbolic tangent function to activate the hidden and output layers. The Gaussian interaction profile core for microbes has been employed to improve binding weights and increase training speed [80].

###### Network consistency projection and multi-data integration

Then Fan et al. combined the matrix created for microbes and diseases with the linear network integration method. Get an integrated similarity matrix for diseases and microbes, and by integrating this matrix, network cohesion prediction was created. Disease-microbe associations were detected by predicting network cohesion and analyzing privileges extracted from them. Human Microbe–Disease Associations Prediction (HMDA-Pred) is a network-based computational method that connects multiple similarity networks to an integrated linear network method and predicts the association of disease-related microbes based on the Network Consistency Projection (NCP) algorithm [81].

###### Link propagation based on node information

PENG et al. have proposed a computational model of node information-based link Propagation for human microbe–disease association prediction (LPHMDA) to prioritize disease-associated microbes. Using well-known associations between disease-causing microbes and similarities between them, the Gaussian interaction profile of the matrix has created a likeness for microbes. They have formed a disease similarity matrix by combining the symptoms of the disease [82].

#### Machine learning-based

**Fig. 3:**
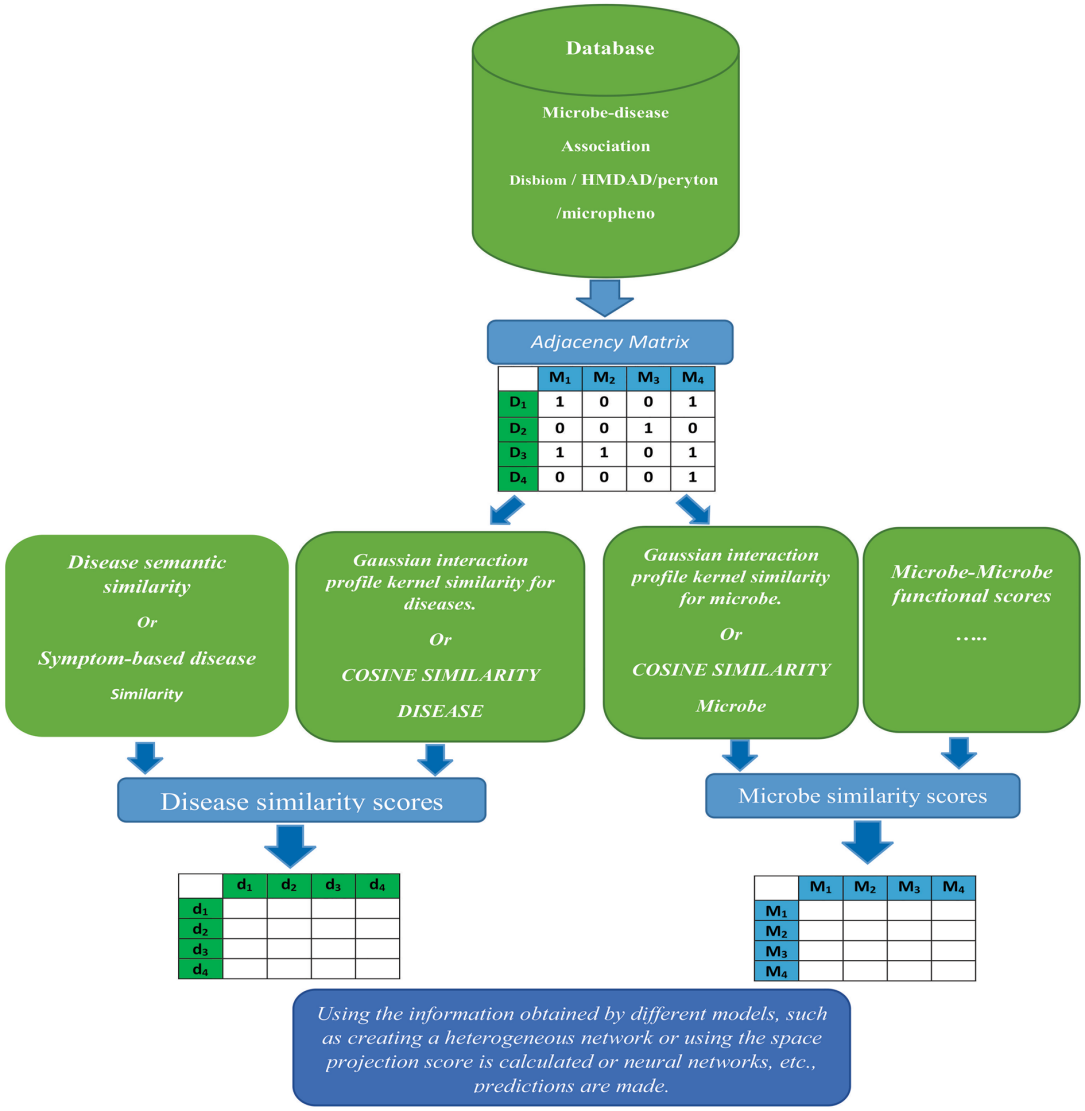
The overall architecture of predicting microbe–disease relationship

Xu et al. proposed a new computational method based on the Kronecker regularized least squares (MDAKRLS) method, which is a machine learning approach, to identify potential associations of microbe–disease communication. To measure the microbial similarity of diseases, they introduced the similarity of the Hamming interaction characteristics. To construct two types of Kronecker similarities between pairs of microbes. Based on the well-known associations, they have calculated the similarity of Kronecker and the similarity of Hamming to disease-microbe pairs. To obtain prediction scores, Kronecker has designed at least four regular squares with different Kronecker similarities. They attained the ultimate forecast outcome by integrating the contributions of distinct similarities [83]. The architecture of predicting the microbe–disease relationship is shown in Fig. 3.

#### Other methods

There are some methods in the literature that do not fit into any of the above-mentioned groups. As a result, these approaches are discussed in this section.

The microbe similarity was calculated from the Gaussian Interaction Profile (GIP) kernel similarity, which is based on the well-known microbe–disease associations. Disease similarity was calculated using the mean of GIP similarity, symptom-based similarity, and functional similarity of the disease. The matrix completion method was used by the Singular Value Threshold (SVT algorithm) to compute the scores of unknown communication between disease-causing microbes. Finally, a low-rank matrix completion(called the MCHMDA) model was proposed [84]. Shi et al. suggest a new predictive method based on the Binary Matrix Completion (called BMCMDA) to forecast possible microbe-noninfectious disease associations (MDAs) by grouping a series of microbe–disease associations into a binary Microbe–disease association matrix. The suggested method suggests that the observed incomplete microbe–disease association matrix is the sum of a latent parameterizing matrix and a noise matrix. It also provides a binomial model for sharing observations that occur independently of the microbial-disease association matrix [85].

The adaptive boosting for human microbe–disease association prediction (ABHMDA) was developed to explore the relationship between diseases and microbes. Due to the lack of sufficient information, the combination of microbial similarity of the GIP kernel and the similarity of symptomatic disease has been considered a feature of the experimental sample. Unknown associations have been used as negative examples as well as positive examples to maintain the balance between the samples during the decision tree training [86]. Lei et al. have proposed a model of microbial disease association with learning graph representations and a modified scoring mechanism on the heterogeneous network (called LGRSH). A heterogeneous network was shaped by combining microbial similarity networks, disease similarity networks, and known microbe–disease associations[87].

#### Recruited datasets and approaches for prediction of microbe–disease associations

Previous studies on the microbial-disease relationship have used a variety of data sources. Table 3 summarises the recruited datasets to predict microbial disease based on the information we reviewed.

**Table 3:** List of all the data that was utilized in the microbe–disease prediction

Data	Source	Original state	Similarity process	URL
HMDAD	The HMDAD database provides documentation of population disorders of disease-related microorganisms in PubMed	HMDAD integrated 483 disease-microbe entries which include 39 diseases and 292 microbes	They're reduced to 450 known MDAs that are then utilized to calculate GIP kernel, Cosine, and Spearman correlation similarity	https://www.cuilab.cn/hmdad
PERYTON	The content of Peryton is entirely supported by the manual curation of biomedical journals. Using reference tools to construct database dictionaries, diseases and Microbiota are supplied in a well-structured, well-organized format	There are currently over 7,900 entries in the database, which link 43 diseases and 1,396 microorganisms	Peryton also provides interactive visualizations, and the data may be downloaded straight to your computer for local storage and analysis	https://dianalab.e-ce.uth.gr/peryton/
GEN-BASED	On DisGeNET, you may find GDAs from UNIPROT, CGI, ClinGen, Genomics England, CTD (human subset), PsyGeNET, Orphanet, and those produced from text mining MEDLINE abstracts	Between 17 549 genes and 24 166 diseases, there are 628 685 GDAs covered. There are 37 diseases mapped, 1850 chromosomes, and 2715 GDAs Size/coverage in HMDAD	The neighbor-based similarity approach calculates GDA scores which were used to find further commonalities among a selection of disorders	https://www.disgenet.org
SYMPTOM-BASED disease data	HSDN pulls data from PubMed's large-scale medical bibliographic records of disease–symptom correlations	Simultaneous counting and TF-IDF weight values for 322 symptoms and 4442 disorders, with 147 97 connections and 22 mapped diseases, 269 symptoms, and 1858 associations of disease symptoms	The symptom-based illness similarity is calculated using Co-occurrence TF-IDFs between one illness and other symptoms	https://www.nature.com/articles/ncomms5212
Semantics-based disease data	MeSH trees are in the National Library of Medicine for a hierarchical definition of disease	Hierarchical trees systematically describe a variety of diseases 33 diseases of size/coverage mapped in HMDAD	The DAG-based semantic similarity of two disease trees made up of hierarchical descriptors is calculated	https://meshb.nlm.nih.gov/search
PROTEIN	STRING is a database that collects protein–protein interactions and data on proteins from several sources	At the species level, 1391 microbes were mapped, with gene neighbor scores of 932 370 pairs of COGs	The neighborhood score is used to determine if there is an edge between two COGs. Also provides interactive visualizations	https://string-db.org
Comprehensive Antibiotic Resistance Database (CARD)	A carefully curated resource offering high-quality reference material on the molecular basis of antimicrobial resistance (AMR), with a focus on the genes, proteins, and mutations implicated in AMR	CARD found 2441 model reference sequences, 853 single nucleotide alterations, as well as an increasing number of indels, frame shift, and nonsense mutations linked to antimicrobial resistance	Additional search criteria include mutations conferring AMR (if relevant) and curated BLAST(P/N) bit score cut-offs are included in the ontology	https://card.mcmaster.ca/
Disbiome	Created in 2018, is a more comprehensive database that is constantly updated every three months	As of December 2019, the Disbiome database includes 322 diseases, 1,470 microbiome organisms, and 9,102 experiments published in 1,018 scholarly articles	The human annotation guarantees a clear and organized presentation of the material that is accessible	https://disbiome.ugent.be/home/
MicroPhenoDB	There are 5677 non-redundant correlations between 1781 microorganisms and 542 human illness phenotypes across more than 22 human body locations in this study	In addition, MicroPhenoDB has 696,934 connections between 27,277 clade-specific core genes and 685 microorganisms	The software allows scientists to search DNA and RNA sequences for potential pathogens without running the usual meta-genomic data processing and assembly steps	http://www.liwzlab.cn/microphenodb

In addition, different approaches for predicting the relationship between microbes and disease are summarized in Table 4.

**Table 4:** Various approaches for predicting the relationship between microbes and diseases

Category	Method	Description
Path-based methods	KATZHMDA, PBHMDA, MDPH_HMDA, BWNMHMDA, WMGHMDA	Numbers and weighted scores of various sorts of pathways between two nodes are often taken into consideration by path-based approaches
Random walk methods	RWRHMDA, BiRWHMDA, PRWHMDA, NTSHMDA, BDSILP, BiRWMP, BRWMDA, NBLPIHMDA, RWHMDA	For iterative walking, random walk algorithms provide a graph-based transition probability matrix
Bipartite local models	LRLSHMDA, NGRHMDA, NCPHMDA, KATZBNRA	BLMs are capable of making independent predictions on both the microbial and disease fronts
Matrix factorization methods	CMFHMDA, GRNMFHMDA, NMFMDA, KBMF, MDLPHMDA, mHMDA	Matrix factorization methods maximize two latent informative matrices, whose multiplication approximates the association matrix with distinct constraint terms, using different constraint terms
Network-based methods	MGATMDA, GATMDA, NINIMHMDA, BPNNHMDA,HMDA-PRED, LPHMDA	Because neural networks can adapt to changing input, they can produce the best possible outcome without requiring the output criteria to be redesigned
Machine learning-based	MDAKRLS	It is a machine learning-based strategy that employs fewer model parameters, saving time and ensuring reliable results
Other methods	ABHMDA, BMCMDA, MCHMDA	Ensemble learning and matrix completion are two of the most common strategies used

### Advantages and disadvantages

The KATZ measure might rebuild probable links concurrently in a vast network, but the computation of GIP kernel similarity will always lead to a bias towards those known relationships. Although the label propagation and random walk algorithms are effective and simple to use, the majority of prediction techniques built on them tend to have less detail. However, when more data is added to the network, training the embeddings will become more challenging. The weighted network-based and heteSim-based methods are excellent at capturing potential subtle semantic associations, but they cannot predict a microbe (drug, disease) in the absence of any known associations. The methods based on matrix factorization can mine deeper potential connections. Matrix factorization has a relatively low spatial complexity because it saves storage space, but selecting the optimal parameters is more challenging. GCN improves the applicability of translation invariance to non-matrix-structured data but it has poor flexibility and scalability. GAT can effectively enhance the aggregation effect of graph neural networks, but it is difficult to aggregate higher-order neighbors. The pooling layer will lose a lot of valuable information and ignore the correlation between the local and the whole.

### Challenges and prospects

Based on the existing studies, some valuable suggestions are provided for further improving predictive performances.

#### Integrating multiple types of data for a single task

In this review, we briefly summarized the advanced and widely used dataset of computational methods related to the problems of microbe–disease and microbe–drug prediction, respectively. To improve prediction performance, the most basic idea is to combine all of these commonly represented databases as a whole to predict any single problem, because they are all closely related In addition, other types of datasets were also introduced, for example, chemical structure-based and phenotype-based data widely used in predictions [88–90], symptom-based disease similarity, and disease semantic similarity in predictions [48, 55]. Certainly, it is a challenge to improve the performance of the prediction model to rationally integrate different types of bioinformatics data to target a prediction task.

#### Introducing new mechanisms

The majority of currently available computational methods improved their performance by enriching more entity similarities than the previous algorithm. In addition to this strategy, many other approaches, such as heterogeneous graph neural network (GCN) and attention mechanisms [91–93], also work for this problem. For example, the attention mechanism can learn the importance of different neighboring nodes and the importance of different node (information) types to a current node. Many GNN models, such as the Spatial Convolution concept [94], can be introduced in link prediction problems. Moreover, most of the existing computational methods are supervised. The limited known associations’ dataset is used as both training and testing sets, which will significantly hinder the utility and performance of the prediction model.

#### Benchmark evaluation

LOOCV and K-fold CV have been widely used as benchmark evaluation frameworks for link predictions. AUROC and ROC plots provide an overview of a predictor's performance and are commonly used to assess the prediction results. The computational approaches developed for the prediction problems of microbe-borne diseases and drugs always use strongly imbalanced datasets. The ROC plots could be misleading when applied in imbalanced prediction scenarios. [95]*.*

#### Handling negative samples

The loss of negative samples significantly affects the prediction performance of the proposed model, and it is crucial to collect negative samples from biomedical databases and literature. To our knowledge, no actual negative samples have been collected and utilized in these predictive tasks presented in this survey. Developing computational methods to generate high-quality negative samples is an alternative to solving this problem [96].

## Available microbiome databases

To the best of our knowledge, three databases have been developed on the subject of microbe–disease interaction, including HMDAD [21], Peryton [97], and Disbiome [98]. Several databases for empirically proven microbe–drug relationships are freely available in the field, such as MDAD [99], abiofilm [100], and Drug Virus.

MDAD (http://chengroup.cumt.edu.cn/MDAD) gathers 5,055 entries containing 1,388 drugs, 180 microbes, and 824 various strains (not including the microbes without defined strains) related to 993 references. All the references were from the 1970s to 2018. We can get all of the codes that correlate here (https://github.com/Sun-Yazhou/MDAD).

The HMDAD (http://www.cuilab.cn/hmdad) is a database that compiles and organizes data on human microbe–disease associations from microbiota investigations. The database-integrated 39 diseases and 292 microbes among the 483 disease-microbe entries.

Peryton (https://dianalab.e-ce.uth.gr/peryton/) is a new database and resource containing empirically supported microbial disease associations. It hosts more than 7900 inputs related to 43 diseases and 1396 microorganisms.

Disbiome (https://disbiome.ugent.be/home/): Created in 2018, is a more comprehensive database that is constantly updated every three months. As of December 2019, the Disbiome database includes 322 diseases, 1,470 microbiome organisms, and 9,102 experiments published in 1,018 scholarly articles.

The ‘a Biofilm’ (http://bioinfo.imtech.res.in/manojk/abiofilm/): There are three sub-categories in this category, including data visualization, a database, and a prediction module. There are 5027 interactions between 1720 medications and 140 microorganisms in the database of anti-biofilm compounds, which includes gram-negative, gram-positive, and fungal microorganisms. Most studies from 1988 to 2017 reported experimental anti-biofilm agents against various microorganisms.

Drug Virus (https://drugvirus.info/tech_doc/) lists the activities and stages of development of 118 drugs that target 83 human viruses. The database allows users to explore virus-BSAA (Broad-spectrum antiviral agents) interactions in real time. DrugVirus.info is a free tool that includes a feedback form on its website. The website will be updated upon request or if a new save-in-man BSAA is discovered or a novel activity of an existing BSAA is discovered.

## Available data based on similarity calculation

This section first discusses the computational method for germ-disease similarities (microbe–drug). It then lists databases and web servers that provide more information about the various diseases, drugs, and microbial components used there.

### Based on disease similarity

*Disease semantic similarity*: Medical records of a particular disease are presented hierarchically in the National Library of Medicine (Mesh). Therefore, to measure the significance of a disease pair, we can use the overlap between the descriptors of the parents. Using both DAGs (directed acyclic graphs), the severity of the disease can be computed [101, 102].

- *Disease symptom similarity*: The similarities of symptomatic diseases based on HSDN were collected by Wheeler et al. [103]. With 849,103 PubMed records, they constructed 147,978 connections between 322 symptoms and 4219 diseases. Based on this data, they derived the similarities based on the symptoms of common diseases from HMDAD [104].
- *Gene-based disease data:* DisGeNET is the largest database on human gene-Disease Association (GDA) and disease types that combines all data in expert-curated repositories, GWAS catalogs, animal models, and scientific articles [105]. MEDLINE is the primary bibliographic database at the National Library of Medicine that holds the number of GDAs [106]. Bravo et al. used HMDAD and GDA databases to calculate the similarity of the recorded diseases.
- *Gene interactions:* The HumanNet v2.0 database (https://www.inetbio.org/humannet/download.php) is now available for efficiently accessing gene interactions, with each interaction having a log-likelihood score (LLS) that assesses the likelihood of a practical linkage between genes [107].

### Based on microbe similarity

*Microbe–microbe interactions*: The MIND database curates the microbe-microbe interaction network data (http://www.microbialnet.org/mind_home.html/) Obtained[39].

- *Microbe data based on protein families*: The STRING database (https://string-db.org) includes protein–protein interactions and protein-related information from a variety of sources. The resource consists of the interactions obtained from computer prediction, information transmission across species, and interactions gathered from other (primary) databases. [52]. The purpose of this database is to achieve a global network of direct and indirect interactions. Collecting, integrating, scoring, and interacting protein-to-protein information, and completing these with computational predictions. Utilizing the proposed method, Kamneva calculated the functional similarity of the microbes [108, 109].
- *Microbe taxonomic similarity*: It contains more than 160,000 species with molecular data in the NCBI database, along with phylogenetic names and lineages and if two microbes have a common progenitor in a certain rank, they have a sequencing likeness to some extent [79].

### Based on drug similarity

- *Drug microbe associations*: Drug Bank (http://www.drugbank.ca/) is a web-based database that contains detailed molecular information about medications, their mechanisms, interactions, and targets. The most recent update was in 2018 [110].
- *Genome sequences:* The NCBI database (https://www.ncbi.nlm.nih.gov/genome/) is used to obtain genome sequences. It contains a wealth of information about the disease that can be used to create DAG charts for disease expression.
- *Heterogeneous networks:* heterogeneous networks, namely microbe–drug heterogeneous networks and microbe–disease–drug networks, from a variety of sources including DrugBank [110], HMDAD [21], and CTD [111].

*Drug structural similarity matrix:* SIMCOMP search service (http://www.genome.jp/tools/simcomp/), is a chart-based solution for finding the most uniformity with the most click-through search on the chart. This server is used to find chemical similarities. The second search server is SUB COMP (http://www.genome.jp/tools/sumcomp/), which is a suggested method for solving the problem of uniformity under graphs. Both of these provide a basis for the study of chemical and physical properties [112].

## Web-based tools

There are several web-based tools available to customize the prediction of microbe–disease associations. Among the web-based tools, there is a Micro Pattern for calculating similarities. For comparison, it divides microorganisms into disease-related classes. Currently, there are no tools available for enrichment analysis of a list of microorganisms. MicroPattern (http://www.cuilab.cn/micropattern) is a web-based tool for microbe set enrichment analysis. [113]. For other areas of expertise MicroPro predicts phenotypes using the complete case and controls frequency profiles and can estimate unknown microbial abundance profiles based on the unplanned readings of metagenomics results (for example, meta PPISP [114], DINIES [115],and DIANA-microT [116] are advanced forecasting methods) [117].Net Cooperate, an online instrument, can measure a host's capacity to provide nutritive support for a parasitic or commensal cell, as well as the (in addition to) complementarity of two microorganisms depending on their metabolic networks [118]. Using web-based operating systems and existing software, a methodological and biomedical study of microorganisms' reactions and humans becomes easier. (http://pharmacomicrobiomics.org) is a research-based online website dedicated to learning about how microbes modulate drug action [119].

## Future directions and conclusion

Machine learning is a very useful technique, and similar algorithms like least squares, matrix factorization, and completion have been commonly applied to problems. Feature-based machine learning algorithms have been hampered by a lack of effective functionality and hence have gained little recognition. In comparison to machine learning, deep learning, which is considered a worthwhile effort, has yet to be implemented in MDA prediction. Many studies have proposed methods based on deep learning that target a complex topological network and catch its node embeddings in response to the aforementioned dilemma. The proposed models in the literature used many types of neural networks to predict drug-microbial communication. Since deep learning methods are a kind of machine learning method, it should be pointed out that their methods could be put to work for further studies to achieve better and more accurate predictions. Considering the ongoing trends of sources, databases, and experimental and laboratory articles in the field of microbiome, medicine, and diseases, more and stronger links between drugs, diseases and microbes could be considered, and forecasting these relationships with the help of computational approaches could pave the way for new microbe-based research discoveries [120–125].

In recent years, significant computational work has been done in the fields of microbes–disease and microbes–drugs. The work done in the field of microbes-disease has been used in different ways than microbes-medicine. Predicting drug and diseases associations with microbiome is very important in revealing the relationship between human diseases and drugs with microbiota. This article provides a thorough examination of forecasting microbial associations. Advanced omics technology and sequencing technologies enable a variety of methods to detect changes in the microbial composition of the patient. Data from existing trials and clinical results are problematic with information loss, non-uniform dispersion, lack of an integrated classification standard, and ambiguity of disease and drugs. To solve these problems using computational methods, machine learning algorithms and especially neural networks are recommended as an inimitable strategy. Machine learning methods are continually evolving, it is believed that the integration and development of these computational algorithms will improve the speed and accuracy of predicting interaction and structure. Most studies and work related to disease and medicine in the field of the microbiome could be conducted with the existence of databases in this field. The specific database for drugs and diseases in the field of the microbiome is very limited and needs to be developed with more experimental entries and accurate computationally predicted entries. Known associations in the field of microbes-disease and microbe–drug are relatively low and this leads to less prediction accuracy. If more links between them are identified and checked experimentally, other interactions will be predicted by computational methods accurately.

## Key points

- Human health is influenced by the microbes that reside within and on human bodies. The microbe–disease association prediction is a computer-based pre-screening tool for clinical trials investigating microorganism-related pathogenic processes.
- Quantitative records of microbial population fluctuation in experimental instances enable the models to conduct fine-grained prediction tasks, and network analysis might become used to infer microbiological pathogenesis with annotated networks of biological events in the future.
- Predicting microbe–drug interactions can assist humans by making medication research and customized therapy efficiently.
- Exploring intricate mechanisms of microorganisms in clinical therapy, drug development, interactions, and repurposing will be considerably aided by prospective microbe–drug relationships prediction.
- .

## Data Availability

Not applicable.

## Abbreviations

LOOCV: Leave-one-out cross validation
AUC: Area under ROC curve
DAG: Directed acyclic graph
HIN: Heterogeneous information network
HMDAD: Human microbe–disease association database
RWR: Random walk with restart
